# Does the Laparoscopic Approach Reduce the Incidence of Vesicourethral Anastomotic Stricture Compared with the Open Approach After Radical Prostatectomy in Patients with Microangiopathic Risk Factors?

**DOI:** 10.3390/medicina62020417

**Published:** 2026-02-22

**Authors:** Alexandru-Ionuț Cherciu, Mihai-Cristian Persu, Andrei-Cosmin Bumbea, Mădălina-Maria Cherciu, Mihnea Cristian Firoiu, Radu Tiberiu Vrabie, Emilian Bolovan, Dragoș Mihail Arbunea, Darius Marian Brînzan, Andreea-Iuliana Ionescu, Radu Dragoș Marcu, Ovidiu-Gabriel Bratu

**Affiliations:** 1 Department of Urology, “Carol Davila” University of Medicine and Pharmacy, 020021 Bucharest, Romania; madalina-maria.budur@drd.umfcd.ro (M.-M.C.); mihnea-cristian.firoiu@drd.umfcd.ro (M.C.F.); radu.vrabie@umfcd.ro (R.T.V.); emilian.bolovan@drd.umfcd.ro (E.B.); dragos-mihail.arbunea@drd.umfcd.ro (D.M.A.); darius-marian.brinzan@umfcd.ro (D.M.B.); marcuradudragos@yahoo.com (R.D.M.); ovidiu.bratu@umfcd.ro (O.-G.B.); 2 Department of Urology, The University Emergency Hospital Bucharest, 050098 Bucharest, Romania; mihai-cristian.persu@rez.umfcd.ro (M.-C.P.); andrei-cosmin.bumbea0625@rez.umfcd.ro (A.-C.B.); 3 Department of Oncological Radiotherapy and Medical Imaging, “Carol Davila” University of Medicine and Pharmacy, 020021 Bucharest, Romania; andreea-iuliana.ionescu@umfcd.ro; 4 Department of Medical Oncology, Colțea Clinical Hospital, 030167 Bucharest, Romania; 5 Department of Urology, Central Military Emergency University Hospital “Dr. Carol Davila”, 010825 Bucharest, Romania

**Keywords:** radical prostatectomy, vesicourethral anastomotic stricture, laparoscopic prostatectomy, open prostatectomy, perioperative risk factors

## Abstract

*Background*: Vesicourethral anastomotic stricture (VUAS) remains a clinically relevant complication following radical prostatectomy, with important implications for postoperative urinary function. Minimally invasive approaches may offer technical advantages; however, their impact on stricture formation in patients with microangiopathic risk factors remains incompletely defined. *Objective*: We aimed to compare the incidence of vesicourethral anastomotic stricture following open radical prostatectomy (ORP) and laparoscopic radical prostatectomy (LRP) in patients with microangiopathic comorbidities and to explore clinical and perioperative factors associated with stricture development. *Materials and Methods*: A retrospective two-centre cohort study was conducted including 115 patients who underwent radical prostatectomy for clinically localized prostate cancer between 2022 and 2024. All patients had at least one microangiopathic risk factor (diabetes mellitus, hypertension, or coronary artery disease). Seventy-two patients underwent ORP and forty-three underwent LRP. VUAS was defined by obstructive symptoms with endoscopic confirmation requiring intervention within 12 months postoperatively. Univariate analyses and exploratory logistic regression models were performed to assess factors associated with stricture formation. *Results*: Vesicourethral anastomotic stricture occurred in 21 patients (18.3%). The crude incidence of VUAS was lower after LRP compared with ORP (9.3% vs. 23.6%); however, this difference did not reach statistical significance. Patients who developed VUAS had a significantly higher body mass index, longer operative time, and greater intraoperative blood loss. In exploratory multivariable analyses, body mass index and operative duration were consistently associated with increased odds of stricture, whereas the effect of surgical approach was unstable and imprecise due to limited event numbers. *Conclusions*: In patients with microangiopathic risk factors, laparoscopic radical prostatectomy was associated with a lower crude incidence of vesicourethral anastomotic stricture compared with open surgery; however, this association was not robust after adjustment. Perioperative and technical factors appear to play a more prominent role in anastomotic healing than surgical approach alone. These findings highlight the importance of optimizing intraoperative conditions to reduce postoperative stricture risk.

## 1. Introduction

Radical prostatectomy (RP) remains one of the principal curative treatment options for patients with localized and selected locally advanced prostate cancer. Advances in surgical technique and perioperative management have led to significant improvements in oncological and functional outcomes; however, postoperative complications continue to represent a relevant clinical burden. Among these, vesicourethral anastomotic stricture (VUAS) is particularly impactful, as it can result in lower urinary tract symptoms, impaired quality of life, and the need for repeated endoscopic or reconstructive interventions [1,2,3].

The reported incidence of VUAS after RP varies widely in the literature, ranging from below 2% in high-volume contemporary minimally invasive series to over 20% in selected cohorts undergoing open surgery or experiencing adverse perioperative conditions [4,5,6,7]. This heterogeneity reflects differences in surgical approach, surgeon experience, anastomotic technique, outcome definitions, and postoperative surveillance strategies [8,9,10]. Historically, open radical prostatectomy (ORP) has been associated with higher stricture rates, whereas minimally invasive approaches, including laparoscopic radical prostatectomy (LRP) and robot-assisted radical prostatectomy (RARP), have demonstrated lower incidences in several contemporary systematic reviews, meta-analyses, and large population-based studies [11,12,13,14]. Improved visualization, reduced tissue manipulation, superior haemostasis, and more precise suturing have been proposed as mechanisms facilitating improved anastomotic healing in minimally invasive surgery [15,16,17].

Despite these observations, the independent effect of surgical approach on VUAS risk remains controversial. Several authors have suggested that the apparent advantages of minimally invasive techniques may be largely mediated by improved perioperative parameters—such as reduced blood loss, shorter operative time, and lower rates of urinary extravasation—rather than by the surgical approach itself [18,19,20]. Moreover, direct comparisons between ORP and LRP remain relatively limited, as many contemporary studies focus predominantly on robotic surgery or combine laparoscopic and robotic techniques into a single minimally invasive category, thereby limiting the interpretability of approach-specific effects [21,22,23].

The pathogenesis of VUAS is multifactorial and involves a complex interaction between local anastomotic factors and systemic patient-related characteristics. From a technical perspective, inadequate mucosal apposition, excessive anastomotic tension, ischemia of the bladder neck or membranous urethra, urinary leakage, and postoperative infection have all been implicated in stricture formation [24,25,26]. Subtle differences in anastomotic technique—such as interrupted versus continuous suturing, the use of barbed versus non-barbed sutures, posterior musculofascial reconstruction, and bladder neck preservation or reconstruction—may further influence anastomotic integrity and long-term patency [27,28,29,30].

In addition to technical considerations, patient-related factors may modulate anastomotic healing. Systemic conditions associated with microvascular dysfunction, including diabetes mellitus, hypertension, and coronary artery disease, are known to impair endothelial function, tissue perfusion, and collagen remodelling, potentially predisposing patients to delayed wound healing and excessive fibrosis [31,32,33]. Obesity has similarly been associated with increased technical complexity, prolonged operative duration, and higher intraoperative blood loss, all of which may adversely affect the local healing environment at the vesicourethral anastomosis [34,35,36]. However, the relative contribution of these systemic factors compared with intraoperative and technical variables remains incompletely understood.

Although multiple studies have evaluated predictors of VUAS following RP, many have analysed heterogeneous patient populations and applied extensive multivariable adjustment without clearly distinguishing confounders from mediators in the causal pathway [37,38,39]. As a result, reported associations between surgical approach and stricture risk have been inconsistent, and interpretation of adjusted estimates has been further complicated by low event rates and differences in modelling strategies [40,41,42]. These limitations highlight the need for focused analyses examining approach-specific outcomes within clinically relevant subgroups.

Patients with microangiopathic risk factors represent a particularly vulnerable population in whom impaired wound healing may exacerbate the risk of anastomotic complications. However, data specifically addressing the impact of surgical approach on VUAS incidence in such patients remain scarce. Clarifying whether laparoscopic surgery is associated with a lower incidence of VUAS in this setting, or whether observed differences are primarily driven by perioperative and technical factors, may have important implications for surgical planning and perioperative optimization.

Therefore, the objective of the present study was to compare the incidence of vesicourethral anastomotic stricture following open and laparoscopic radical prostatectomy in patients with microangiopathic risk factors and to explore clinical and perioperative variables associated with postoperative stricture development.

## 2. Materials and Methods

### 2.1. Study Design and Reporting Standards

This study was designed as a retrospective, two-centre cohort analysis comparing open radical prostatectomy (ORP) and laparoscopic radical prostatectomy (LRP) in patients with microangiopathic risk factors. The study was conducted in accordance with the Strengthening the Reporting of Observational Studies in Epidemiology (STROBE) guidelines for cohort studies.

### 2.2. Study Setting and Patient Selection

We retrospectively reviewed consecutive patients who underwent radical prostatectomy for clinically localized prostate adenocarcinoma between January 2022 and December 2024 at two urology units: a public tertiary referral hospital and a private surgical clinic. The two institutions functioned as parallel clinical settings in which the same surgical team performed procedures during the study period. Thus, differences between centres reflected administrative and patient referral pathways rather than differences in surgical personnel or operative philosophy.

Surgical approach (ORP or LRP) was determined based on surgeon expertise, anatomical considerations, and patient preference after preoperative counselling; no randomization was performed. The distribution of surgical approaches across the two centres was influenced primarily by patient choice and institutional logistics rather than by differences in surgical skill or technique, as the operating surgeons were identical in both settings.

Patients were eligible for inclusion if they met all the following criteria:

- Histologically confirmed prostate adenocarcinoma on postoperative paraffin-embedded specimens.
- Radical prostatectomy performed using either an open retropubic or laparoscopic approach.
- Availability of complete perioperative and follow-up data.
- Presence of at least one microangiopathic risk factor, defined as diabetes mellitus, hypertension, or coronary artery disease under active medical treatment at the time of surgery.

Exclusion criteria were:

- Loss to follow-up within 12 months postoperatively.
- Incomplete clinical or operative records.
- History of prior urinary tract pathology or surgery affecting the bladder neck or urethra (including previous transurethral resection of the prostate, urethral stricture disease, or bladder cancer).

### 2.3. Definition and Assessment of Outcome

The primary endpoint of the study was the occurrence of vesicourethral anastomotic stricture (VUAS) within 12 months following surgery. VUAS was defined as the presence of obstructive lower urinary tract symptoms associated with endoscopic confirmation of an anastomotic narrowing that prevented passage of a cystoscope and required endoscopic intervention (dilation or incision). All diagnoses were confirmed by cystoscopy.

Postoperative follow-up was standardized across both centres and consisted of outpatient evaluations at 3, 6, 9, and 12 months, including assessment of voiding symptoms and urinary continence. Cystoscopic evaluation was performed in symptomatic patients; routine surveillance cystoscopy in asymptomatic individuals was not employed.

### 2.4. Surgical Technique

All procedures were performed by high-volume urologists experienced in oncologic pelvic surgery. Patients were positioned supine with Trendelenburg tilt. Following removal of the prostate and seminal vesicles, vesicourethral reconstruction was performed according to institutional standards.

In open radical prostatectomy, the vesicourethral anastomosis was typically constructed using interrupted absorbable sutures placed circumferentially to achieve mucosa-to-mucosa apposition. Bladder neck calibration was performed as needed to achieve a diameter of approximately 20–22 Fr, with routine mucosal eversion.

In laparoscopic radical prostatectomy, the anastomosis was generally performed using a continuous running suture technique, most commonly with a 3-0 barbed suture (V-Loc™), allowing for uniform tension distribution and watertight closure. Posterior reconstruction was performed at the surgeon’s discretion and was not formally standardized across centres.

In all cases, reconstruction was performed over a 20–22 Fr transurethral catheter. Ilio-obturator pelvic lymphadenectomy was routinely performed, with similar indications across surgical approaches. One or two pelvic drains were placed according to intraoperative judgement. The urethral catheter was maintained for 10 days, with cystography or pericatheter urethrography performed prior to catheter removal to confirm anastomotic integrity.

### 2.5. Assessment of Covariates

Baseline demographic and clinical variables included age, body mass index (BMI), preoperative prostate-specific antigen (PSA) level, smoking status, and comorbidities. Diabetes mellitus, hypertension, and coronary artery disease were recorded as present if documented in the medical record and under active treatment at the time of surgery.

Perioperative variables included operative time (minutes), estimated blood loss (millilitres), and length of hospitalization (days). Operative time and blood loss were treated as continuous variables and analysed using clinically meaningful increments.

### 2.6. Statistical Analysis

Continuous variables were assessed for normality using the Shapiro–Wilk test and are reported as mean ± standard deviation or median with interquartile range, as appropriate. Group comparisons were performed using the independent-samples *t*-test or Mann–Whitney U test for continuous variables and the chi-square or Fisher’s exact test for categorical variables.

To explore factors associated with VUAS, logistic regression analyses were conducted with an explicitly exploratory intent due to the limited number of outcome events. The primary analytical focus was placed on univariate associations and a parsimonious multivariable model to reduce the risk of overfitting and sparse-data bias.

The initial model evaluated the crude association between surgical approach and VUAS. A secondary model included surgical approach and selected perioperative variables (BMI and operative time), chosen a priori based on biological plausibility and prior literature. Operative time was analysed per 10-min increase, and blood loss per 100 mL increase. Perioperative variables considered potential mediators of the effect of surgical approach were not included in models intended to estimate the total effect of approach, to avoid overadjustment.

As a complementary exploratory approach, penalized logistic regression models using the least absolute shrinkage and selection operator (LASSO) was fitted to further evaluate factors associated with VUAS. Given the limited number of outcome events, penalization was applied to reduce overfitting and improve model stability. Predictors were standardized prior to model fitting, and the penalty parameter (λ) was selected via 10-fold cross-validation using the one–standard-error rule to favour a more parsimonious model. This analysis was performed using the glmnet package in R (Software Version 4.5.2.).

Adjusted odds ratios (ORs) with 95% confidence intervals (CIs) are reported. Statistical significance was defined as a two-sided *p* value < 0.05. All analyses were performed using JASP software (Version 0.95.2), with penalized regression analyses conducted in R.

### 2.7. Ethical Considerations

The study was conducted in accordance with the principles of the Declaration of Helsinki. Ethical approval was obtained independently from the institutional ethics committees of both participating centres for observational studies involving patients undergoing surgical treatment for prostate cancer.

All patients provided written informed consent at the time of hospital admission and prior to surgery. This consent included agreement to undergo the surgical procedure as well as permission for the use of their clinical and perioperative data for scientific and research purposes, including retrospective observational studies. No additional study-specific informed consent was required for the present analysis, as the research involved secondary use of data collected as part of routine clinical care.

## 3. Results

### 3.1. Baseline Patient Characteristics and Surgical Approach

**Figure 1:**
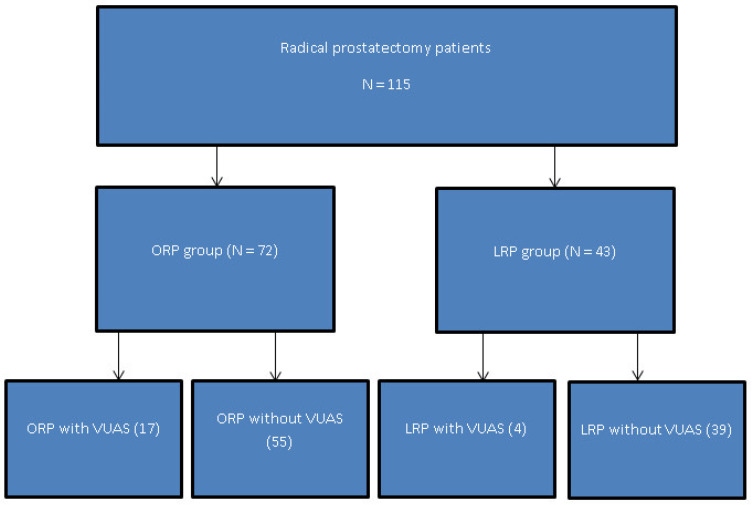
Flow diagram of patient allocation to open radical prostatectomy (ORP) and laparoscopic radical prostatectomy (LRP), with subsequent development of vesicourethral anastomotic stricture (VUAS).

A total of 115 patients met the inclusion criteria and were included in the final analysis, with 72 patients undergoing open radical prostatectomy (ORP) and 43 patients undergoing laparoscopic radical prostatectomy (LRP). Patient selection, exclusion, and allocation according to surgical approach, together with the subsequent occurrence of vesicourethral anastomotic stricture (VUAS), are detailed in the flow diagram (Figure 1).

Baseline demographic and clinical characteristics stratified by surgical approach are summarized in Table 1. The two groups were comparable in terms of age (64.1 ± 6.0 years in the ORP group vs. 64.8 ± 6.2 years in the LRP group; *p* = 0.643) and preoperative prostate-specific antigen (PSA) levels (14.6 ± 5.8 ng/mL vs. 13.8 ± 5.3 ng/mL; *p* = 0.730). Patients treated with ORP had a significantly higher mean body mass index (BMI) compared with those undergoing LRP (27.7 ± 3.1 kg/m^2^ vs. 26.0 ± 2.8 kg/m^2^; *p* < 0.001).

**Figure 2:**
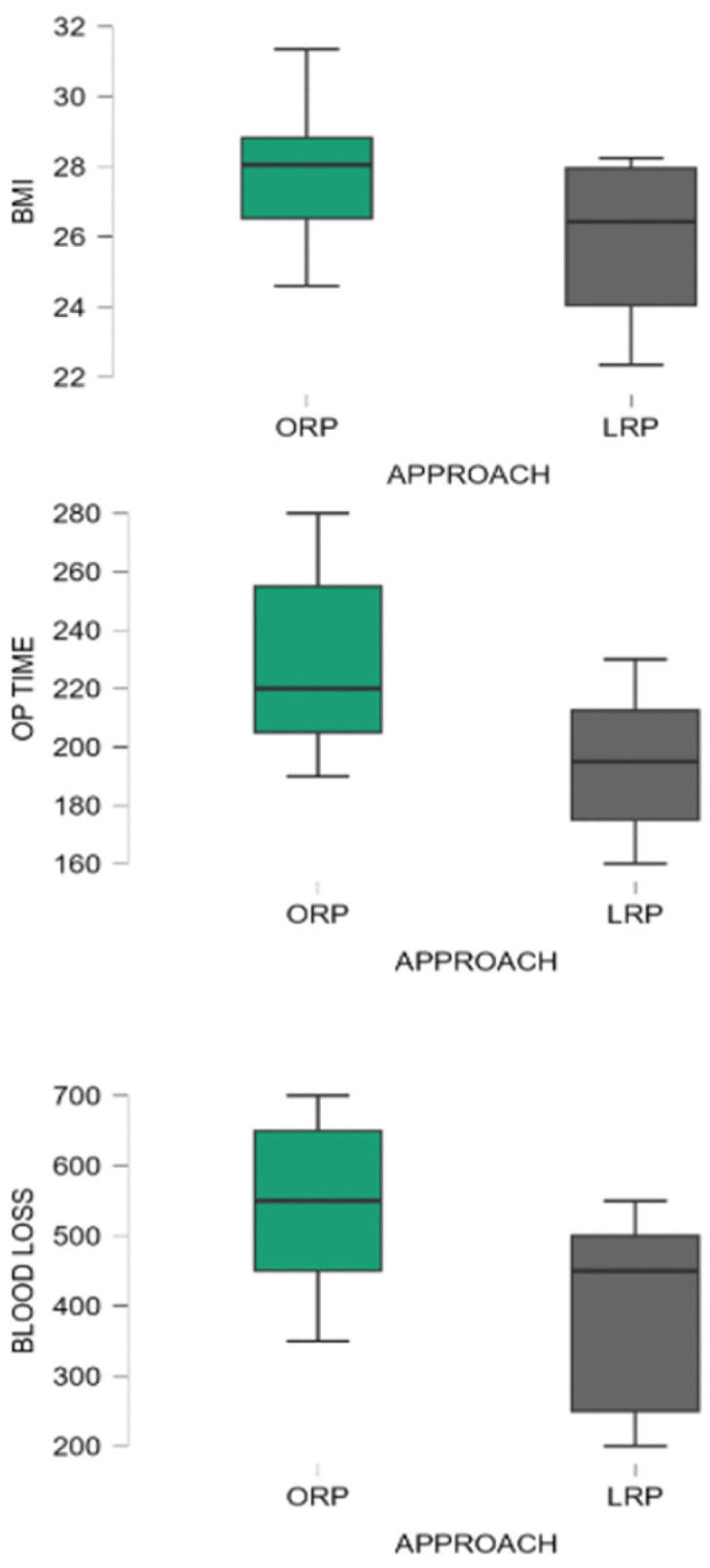
Boxplots showing distribution of body mass index (BMI), operative time, and intraoperative blood loss according to surgical approach. ORP = open radical prostatectomy; LRP = laparoscopic radical prostatectomy.

Significant differences were also observed in perioperative parameters. ORP was associated with longer operative duration (230.5 ± 25.1 min vs. 193.7 ± 20.4 min; *p* < 0.001), greater estimated intraoperative blood loss (536 ± 120 mL vs. 400 ± 80 mL; *p* < 0.001), and prolonged length of hospitalization (6.5 ± 1.2 days vs. 4.9 ± 0.8 days; *p* < 0.001). The distribution of BMI, operative time, and intraoperative blood loss according to surgical approach is illustrated in Figure 2.

The prevalence of microangiopathic comorbidities, including diabetes mellitus, hypertension, and coronary artery disease, as well as smoking history, did not differ significantly between the two surgical groups (all *p* > 0.40).

### 3.2. Incidence of Vesicourethral Anastomotic Stricture

Overall, vesicourethral anastomotic stricture (VUAS) developed in 21 of the 115 patients, corresponding to an incidence of 18.3% within 12 months after radical prostatectomy.

**Figure 3:**
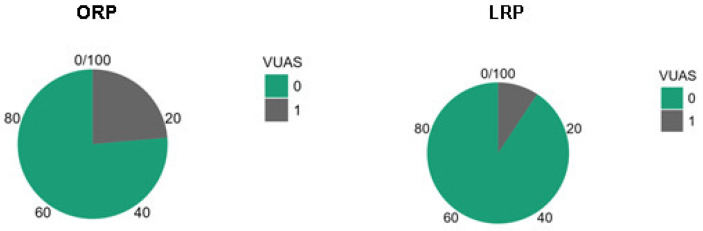
Incidence of vesicourethral anastomotic stricture (VUAS) according to surgical approach. ORP = open radical prostatectomy; LRP = laparoscopic radical prostatectomy.

The incidence of VUAS differed according to surgical approach (Figure 3). VUAS occurred in 17 of 72 patients (23.6%) following ORP and in 4 of 43 patients (9.3%) following LRP. The absolute difference in stricture rates between approaches was 14.3 percentage points. Although the incidence was numerically lower in the laparoscopic group, this difference did not reach statistical significance in unadjusted comparison (*p* = 0.063).

### 3.3. Univariate Analysis

Univariate comparisons between patients who developed VUAS and those who did not revealed significant associations with several perioperative and patient-related variables. Patients with VUAS had a significantly higher BMI (30.8 ± 2.9 kg/m^2^ vs. 26.2 ± 2.7 kg/m^2^; *p* < 0.001), longer operative time (253 ± 24 min vs. 202 ± 21 min; *p* < 0.001), and greater estimated blood loss (703 ± 115 mL vs. 447 ± 92 mL; *p* < 0.001).

No statistically significant differences were observed with respect to age, preoperative PSA level, diabetes mellitus, hypertension, coronary artery disease, or smoking status (all *p* > 0.10).

### 3.4. Multivariable Analysis

To further explore factors associated with the development of VUAS, a series of logistic regression models were constructed, with explicit recognition of the limited number of outcome events and the resulting risk of model instability.

In the crude model evaluating surgical approach alone (Model 1), laparoscopic radical prostatectomy was associated with a lower odd of VUAS compared with open surgery (odds ratio [OR] 0.33, 95% confidence interval [CI] 0.10–1.06); however, this association did not reach statistical significance (*p* = 0.063) (Table 2).

**Figure 4:**
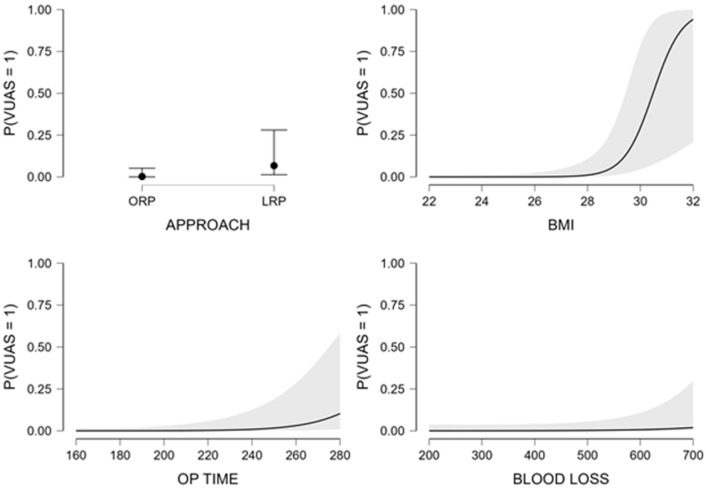
Adjusted odds ratios with 95% confidence intervals for predictors of vesicourethral anastomotic stricture (VUAS) derived from multivariable logistic regression (Model 2). ORP = open radical prostatectomy; LRP = laparoscopic radical prostatectomy.

**Figure 5:**
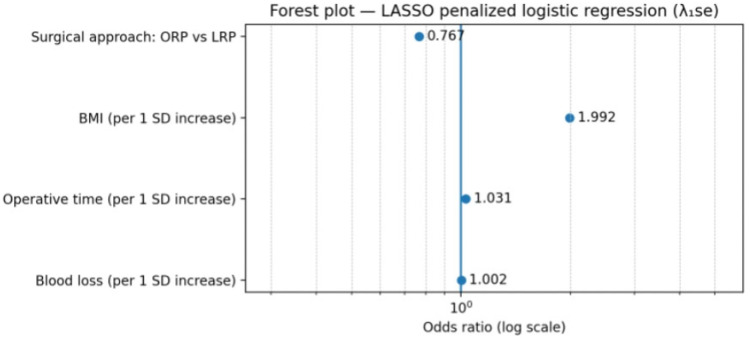
Forest plot of odds ratios from the LASSO-penalized logistic regression model (Model 2) (λ_1_se). Points represent odds ratios on a logarithmic scale. Continuous predictors are scaled per one standard deviation increase.

In the primary adjusted model (Model 2), which included surgical approach together with selected perioperative variables (BMI, operative time, and estimated blood loss), higher BMI and longer operative duration were independently associated with increased odds of VUAS. The adjusted odds ratios and 95% confidence intervals for all predictors included in this model are presented in Table 3 and illustrated graphically in Figure 4. In this model, the estimated effect of surgical approach showed substantial imprecision, with wide confidence intervals, reflecting the small number of VUAS events in the laparoscopic group. The penalized model retained surgical approach, body mass index, operative time and blood loss as predictors with non-zero coefficients. The resulting odds ratios are shown in Table 3 and are illustrated graphically in Figure 5. All reported odds ratios for continuous variables correspond to a one standard deviation increase due to internal standardization. Penalized models do not provide conventional *p*-values or confidence intervals.

**Figure 6:**
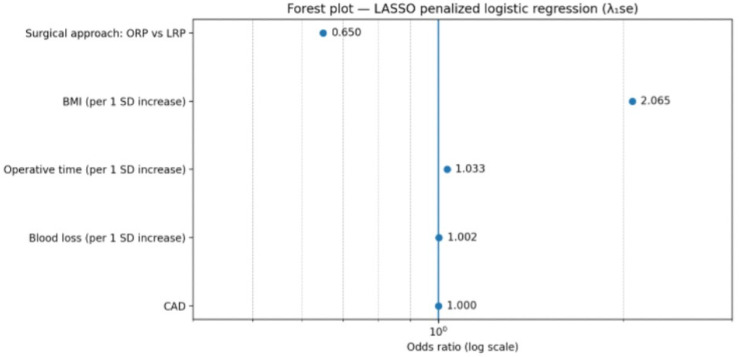
Forest plot of odds ratios from the LASSO-penalized logistic regression model (Model 3) (λ_1_se). Points represent odds ratios on a logarithmic scale. Continuous predictors are scaled per one standard deviation increase.

A secondary adjusted model incorporating coronary artery disease as an additional covariate (Model 3) yielded comparable results (Table 4). BMI and operative time remained consistently associated with VUAS, whereas coronary artery disease did not demonstrate an independent association. The direction and magnitude of the surgical approach coefficient were similar to those observed in Model 2, although confidence intervals remained wide. The penalized model retained surgical approach, body mass index, operative time and blood loss as predictors with non-zero coefficients, while coronary artery disease was not retained after penalization. The resulting odds ratios are shown in Table 4 and are illustrated graphically in Figure 6. All reported odds ratios for continuous variables correspond to a one standard deviation increase due to internal standardization. Penalized models do not provide conventional *p*-values or confidence intervals.

Given that operative time and blood loss are plausibly downstream consequences of surgical approach and technical complexity, multivariable findings were interpreted cautiously and considered exploratory rather than confirmatory.

### 3.5. Summary of Results

In summary, laparoscopic radical prostatectomy was associated with a lower crude incidence of vesicourethral anastomotic stricture compared with open surgery. However, this difference did not reach statistical significance, and multivariable analyses suggested that patient-related and perioperative factors—particularly body mass index and operative duration—were more consistently associated with stricture development than surgical approach alone. The attenuation and reversal of the surgical approach effect after adjustment for these variables suggest that differences in operative characteristics may contribute to the observed variation in VUAS risk, consistent with mediation through operative factors rather than a protective effect of surgical approach.

## 4. Discussion

Vesicourethral anastomotic stricture remains a clinically relevant complication following radical prostatectomy, with important implications for postoperative urinary function and quality of life. In the present retrospective two-centre cohort, the crude incidence of VUAS was lower after laparoscopic radical prostatectomy compared with open surgery; however, this difference did not reach statistical significance. Exploratory multivariable analyses suggested that patient- and procedure-related factors, particularly body mass index and operative duration, were more consistently associated with stricture development than surgical approach alone.

The overall incidence of VUAS observed in this study (18.3%) is higher than that reported in several contemporary series, particularly those dominated by robot-assisted radical prostatectomy [1,12,14]. This discrepancy is likely multifactorial. First, our cohort was intentionally enriched for patients with microangiopathic risk factors, a population in whom impaired tissue perfusion and delayed wound healing may predispose to anastomotic complications [22,23]. Second, stricture diagnosis in the present study was symptom-driven but systematically confirmed endoscopically, potentially increasing detection compared with studies relying solely on administrative codes or intervention rates [2]. Finally, institutional and surgeon-related factors, including technique heterogeneity and postoperative management pathways, may also contribute to variability in reported stricture rates [15,19].

Minimally invasive approaches have consistently demonstrated lower rates of vesicourethral anastomotic stricture in large contemporary series, particularly in the context of robot-assisted radical prostatectomy [1,12,13]. Proposed mechanisms include enhanced visualization, improved precision during anastomotic suturing, and reduced tissue trauma. However, accumulating evidence suggests that these advantages may be largely mediated through improved perioperative conditions—such as reduced blood loss and shorter operative times—rather than representing an independent protective effect of surgical approach per se [15,20,21]. Our findings are concordant with this interpretation, as adjustment for perioperative variables resulted in unstable estimates for surgical approach, with wide confidence intervals reflecting sparse event numbers in the laparoscopic group.

Body mass index emerged as a consistent predictor of VUAS across univariate and multivariable analyses. Obesity is known to increase technical complexity during pelvic surgery, often resulting in reduced exposure, increased anastomotic tension, and prolonged operative duration, all of which may adversely affect anastomotic healing [17,18,34,36]. Similarly, longer operative time likely reflects increased surgical difficulty and prolonged tissue manipulation, factors previously associated with higher rates of postoperative complications, including anastomotic stricture [3,25]. These findings reinforce the concept that technical execution and intraoperative conditions play a central role in determining anastomotic outcomes.

Interestingly, microangiopathic comorbidities such as diabetes mellitus, hypertension, and coronary artery disease were not independently associated with VUAS in adjusted analyses. While these conditions are mechanistically linked to impaired microvascular perfusion and delayed wound healing, their impact may be attenuated in patients receiving contemporary medical management or may be overshadowed by stronger intraoperative determinants of anastomotic integrity [23,33]. Alternatively, the limited sample size and absence of detailed markers of disease severity (e.g., glycemic control or end-organ damage) may have reduced the ability to detect independent associations.

Robot-assisted radical prostatectomy was not directly evaluated in this study but warrants discussion considering existing literature. Multiple recent meta-analyses and large population-based studies have demonstrated lower stricture rates following robotic surgery compared with both open and laparoscopic approaches [12,13]. These findings likely reflect not only technological advantages but also higher procedure volumes, standardized anastomotic techniques, and structured postoperative pathways in robotic centres. In this context, surgical approach may act as a surrogate marker for technical refinement rather than as an isolated causal factor.

Several limitations should be acknowledged. The retrospective design introduces inherent risks of selection bias and residual confounding, particularly in terms of centre- and surgeon-specific factors that could not be fully accounted for. The number of VUAS events, especially in the laparoscopic group, was limited, constraining the stability of multivariable models and necessitating cautious interpretation of adjusted estimates. Additionally, the follow-up period was limited to 12 months and may have underestimated late-presenting strictures. Despite these limitations, the consistency of associations observed for BMI and operative duration, together with their biological plausibility and concordance with prior literature, strengthens the credibility of the findings.

Overall, the present study supports the concept that perioperative and technical factors play a dominant role in vesicourethral anastomotic healing following radical prostatectomy. While laparoscopic surgery was associated with a lower crude incidence of VUAS, this apparent advantage appears to be mediated primarily through improved operative parameters rather than representing an independent protective effect of surgical approach.

## 5. Conclusions

In this retrospective two-centre cohort of patients with microangiopathic risk factors, laparoscopic radical prostatectomy was associated with a lower crude incidence of vesicourethral anastomotic stricture compared with open surgery. However, this difference did not reach statistical significance, and exploratory multivariable analyses demonstrated substantial imprecision in estimates related to surgical approach.

Across all analyses, patient- and procedure-related factors—particularly body mass index and operative duration—were more consistently associated with the development of vesicourethral anastomotic stricture than surgical approach alone. These findings support the concept that intraoperative conditions and technical complexity play a dominant role in anastomotic healing following radical prostatectomy.

While minimally invasive surgery may provide a more favourable operative environment, its apparent benefit with respect to stricture formation appears to be largely mediated through improved perioperative parameters rather than representing an independent protective effect. Future prospective studies with larger sample sizes, standardized surgical techniques, and longer follow-up are required to further clarify the relative contributions of patient characteristics, operative factors, and surgical approach to vesicourethral anastomotic outcomes.

## Figures and Tables

**Table 1:** Baseline Patient Characteristics by Surgical Approach.

Characteristic	ORP (n = 72)	LRP (n = 43)	*p*-Value
Age (years)	64.1 ± 6.0	64.8 ± 6.2	0.643
PSA (ng/mL)	14.6 ± 5.8	13.8 ± 5.3	0.730
BMI (kg/m^2^)	27.7 ± 3.1	26.0 ± 2.8	<0.001
Operative time (min)	230.5 ± 25.1	193.7 ± 20.4	<0.001
Blood loss (mL)	536 ± 120	400 ± 80	<0.001
Hospitalization (days)	6.5 ± 1.2	4.9 ± 0.8	<0.001
Diabetes mellitus, n (%)	21 (29.2%)	12 (27.9%)	0.876
Hypertension, n (%)	30 (41.7%)	18 (41.9%)	0.982
Coronary artery disease, n (%)	19 (26.4%)	13 (30.2%)	0.657
Smoking history, n (%)	25 (34.7%)	18 (41.9%)	0.437
VUAS, n (%)	17 (23.6%)	4 (9.3%)	0.063 (crude)

**Table 2:** Model 1—Crude Model (Approach Only).

Variable	OR	95% CI	*p*-Value
LRP vs. ORP	0.33	0.10–1.06	0.063

**Table 3:** Model 2—Primary Adjusted Model.

Variable	OR	95% CI	*p*-Value
LRP vs. ORP	35.47	2.44–515.51	0.009
BMI	6.21	1.70–22.60	0.006
Operative time (per min)	1.066	1.025–1.109	0.001
Blood loss (per mL)	1.011	0.998–1.024	0.102
Model 2—Primary Adjusted Model (Penalized)			
Variable	OR		
ORP vs. LRP	0.77		
BMI	1.99		
Operative time (per min)	1.03		
Blood loss (per mL)	1.00		

**Table 4:** Model 3—Primary Adjusted Model (+CAD).

Variable	OR	95% CI	*p*-Value
LRP vs. ORP	34.13	2.314–503.468	0.010
BMI	6.50	1.690–25.017	0.006
Operative time (per min)	1.066	1.025–1.109	0.002
Blood loss (per mL)	1.010	0.998–1.023	0.112
CAD	1.255	0.239–6.596	0.788
Model 3—Primary Adjusted Model (+CAD) (Penalized)			
Variable	OR	Retained in model	
ORP vs. LRP	0.65	Yes	
BMI	2.06	Yes	
Operative time (per min)	1.03	Yes	
Blood loss (per mL)	1.00	Yes	
CAD	1.00	Yes	

## Data Availability

The original contribution presented in this study is included in the article. Further inquiries can be directed to the corresponding author.

## Abbreviations

The following abbreviations are used in this manuscript:

VUAS	Vesicourethral anastomotic stricture
ORP	Open radical prostatectomy
LRP	Laparoscopic radical prostatectomy
RP	Radical prostatectomy
RARP	Robot-assisted radical prostatectomy
RRP	Retropubic radical prostatectomy
PCa	Prostate cancer
TURP	Transurethral resection of the prostate
ORs	Odds ratios
CIs	Confidence intervals
BMI	Body mass index
PSA	Prostate-specific antigen
CAD	Coronary artery disease
